# Evaluation of Apple Maturity with Two Types of Dielectric Probes

**DOI:** 10.3390/s18010121

**Published:** 2018-01-04

**Authors:** Marcin Kafarski, Andrzej Wilczek, Agnieszka Szypłowska, Arkadiusz Lewandowski, Piotr Pieczywek, Grzegorz Janik, Wojciech Skierucha

**Affiliations:** 1 Institute of Agrophysics, Polish Academy of Sciences, Doświadczalna 4, 20-290 Lublin, Poland; a.wilczek@ipan.lublin.pl (A.W.); a.szyplowska@ipan.lublin.pl (A.S.); p.pieczywek@ipan.lublin.pl (P.P.); w.skierucha@ipan.lublin.pl (W.S.); 2 Institute of Electronic Systems, Warsaw University of Technology, Nowowiejska 15/19, 00-665 Warsaw, Poland; a.lewandowski@elka.pw.edu.pl; 3 Institute of Enviromental Protection and Development, Wroclaw University of Environmental and Life Sciences, Pl. Grunwaldzki 24, 50-363 Wrocław, Poland; grzegorz.janik@upwr.edu.pl

**Keywords:** dielectric probe, apple shelf-life, dielectric dispersion

## Abstract

The observed dielectric spectrum of ripe apples in the last period of shelf-life was analyzed using a multipole dielectric relaxation model, which assumes three active relaxation processes: primary α-process (water relaxation) and two secondary processes caused by solid-water-ion interactions α’ (bound water relaxations), as well as β’ (Maxwell-Wagner effect). The performance of two designs of the dielectric probe was compared: a classical coaxial open-ended probe (OE probe) and an open-ended probe with a prolonged central conductor in a form of an antenna (OE-A-probe). The OE-A probe increases the measurement volume and consequently extends the range of applications to other materials, like granulated agricultural products, soils, or liquid suspensions. However, its measurement frequency range is limited as compared to the OE probe because, above 1.5 GHz, the probe with the antenna generates higher propagation modes and the applied calibrations and calculations are not sufficient. It was shown that data from measurements using the OE-A probe gave slightly stronger correlations with apples’ quality parameters than using the typical OE probe. Additionally, we have compared twelve multipole fitting models with different combinations of poles (eight three-pole and four two-pole models). It was shown that the best fit is obtained using a two-pole model for data collected for the OE-A probe and a three-pole model for the OE probe, using only Cole-Cole poles in both cases.

## 1. Introduction

The interest in dielectric properties of agricultural products is driven by a practical need for the development of fast and non-destructive techniques for the measurement of vital quality properties such as moisture, chemical composition, structure, texture, etc. [1]. Direct determination of these properties of agricultural products is either difficult, laborious, expensive, or destructive to the samples [2]. Dielectric measurements that can be performed using a variety of time- and frequency-domain techniques proved to be fast, relatively inexpensive in comparison to chemical analyses, easily adaptable to automatic monitoring applications, and non-destructive or minimally invasive to the tested material [3,4,5,6,7,8,9,10]. Especially, water content is an example of a vital property that is easily measured using dielectric sensors [4,6,11]. 

In the case of apples, there is a need to determine the optimal harvesting time and to monitor their quality during storage using non-destructive means. Microwave dielectric spectroscopy techniques could be potentially applied for this purpose, either alone or in combination with other non-destructive methods, such as biospeckle activity monitoring [12]. However, the development of such techniques requires the detailed knowledge of broadband dielectric properties of apples, the distribution of dielectric permittivity in the volume of an apple, and the determination of specific factors influencing the dielectric permittivity in given frequency ranges.

Broadband dielectric response of apples was examined by many researchers. Bhosale et al. [13] predicted the firmness of apples during shelf life using of a parallel plate sensor at low frequencies: 132, 640, 880 kHz. Guo et al. [14] measured 10–18,000 MHz dielectric spectrum of the external surface and the interior tissue of three apple cultivars at 24 °C over 10 weeks of storage at 4 °C and correlated the results with quality factors of apples: soluble solids content (SSC), firmness, moisture content, and pH. Although a high correlation was observed in a linear relationship between the dielectric constant divided by SSC and the dielectric loss factor divided by SSC in the complex plane, the SSC was not predicted well from that relationship, and no high correlations were found between the dielectric properties and SSC, moisture content, firmness, or pH. The dielectric constant and loss factor remained essentially constant during the 10-week storage period. Castro-Giráldez et al. [15] developed a dielectric spectroscopy technique from 500 MHz to 20 GHz for the determination of apple (Granny Smith cultivar) maturity in the form of a newly-defined dielectric maturity index (MI) as the difference between the loss factors at the dipolar relaxation frequency and at 0.5 GHz. The choice of selected frequencies was used due the fact that apple maturation produces an increase in the sugar content (decreasing loss factor at relaxation frequency) and an increase in the malate content (increasing loss factor at 0.5 GHz). The authors of [16] tested dielectric properties of the external surface, internal tissue and the juice of Fuji apples during the last two months of tree-ripening. The apples were measured with an open-ended coaxial-line probe and a network analyzer at 24 °C from 0.01 to 4.5 GHz. No obvious correlations were found between permittivity and firmness, moisture content or pH. They concluded that further studies were needed to assess the potential usefulness of dielectric properties for sensing the apple maturity or internal quality. A report on dielectric constants and loss factors of apples with skin and without skin, and flesh juice of the same Fuji apples cultivar, during 21-week storage at 5 °C and measured at a three-week interval and 24 °C from 0.01 to 4.5 GHz was presented in [17]. The pH increased and firmness decreased with storage time. There was no obvious change or trend in the moisture content, SSC, conductivity, and permittivity during storage. The depths of penetration in skin-on and skin-off apples, and apple juice decreased with increased frequency. Weak correlation between the apple dielectric permittivity and the internal qualities and limited penetration depth shows that sensing apple internal qualities from permittivity of a skin-on apple, a skin-off apple and the apple juice is difficult. In conclusions the authors stressed weak correlations between permittivity and quality indices, which suggest that sensing the apple quality from their dielectric properties might not be practical under experimental conditions.

It seems that broadband dielectric measurement of apples is not sufficiently effective in the form of producing a reliable quality index, especially before and at early stage of maturity. The results presented in [15] are quite promising but the destructive measurements presented in [15] were performed in the senescence period of apples, in which they are losing their commercial value. 

The objectives of research presented in this paper were to: (1) test the performance of two types of open-ended dielectric probes, with (OE-A) and without (OE) an antenna in the frequency range 10 MHz–20 GHz using finite element method (FEM) digital simulations, vector network analyzer (VNA) measurements of liquid reference materials (distilled water, ethanol, and methanol), and apples; and (2) test the change of dielectric properties of ripe apples at the time when they are starting to lose their commercial value and possibly determine the dielectric indicators of apples at the time of reaching the final stage of their commercial value by correlating the measured physicochemical parameters with the parameters of the multipole dielectric model.

The frequency range of 10 MHz–20 GHz was chosen to cover all frequency ranges presented in available literature and the 20 GHz frequency limit was important for observing free water dielectric effect in apples. In other similar studies apples have never been measured using OE-A (only with a typical OE, coaxial-line probe).

## 2. Materials and Methods

### 2.1. Apples and Reference Liquids

The measurements were done in laboratory conditions with a controlled temperature of 20 ± 1 °C. The air humidity was 50% ± 10%. The tested apples were of the Janagold Decosta variety. They were collected from Stryjno Sad cold store near Lublin, Poland, were they were kept in 2–4 °C before distribution to local grocery shops. The harvesting time of this variety was the beginning of October and the maximum storage time is four months in cold store conditions. Thus, the measurement time (end of January) was intentionally chosen at the end of their commercial value when they quickly become ripe in room conditions.

After dielectric measurements every apple from each series was tested to determine its firmness and crispness as well as the soluble solid content. Apple firmness (as the maximum force inserted to the apple flesh by the puncture probe) and crispness (as the number of acoustic events when puncturing an apple) were measured using two methods: standard puncture test and acoustic emission described in detail in [18]. Soluble solids content as the measure of sugars, organic and amino acids, and soluble pectins in fruit juice was measured for each punctured apple using a pocket digital refractometer (PAL-BX/RI by Atago CO., LTD., Tokyo, Japan). The refractometer provided measurements in the Brix scale with a high measurement accuracy of ±0.1%. (the refractive index measurement resolution −0.0001). Measuring procedure consisted of extraction of juice from apples parenchyma tissue followed by filtering through a paper filter. Small, 90 µL doses of the filtered apple juice were dropped on the refractometer by use of an automatic pipette.

The reference values for the relative dielectric permittivity of water, ethanol and methanol used for the calibration of the sensors were calculated from the Cole-Cole formula [19]:

(1) $$\varepsilon_{r}^{*}=\varepsilon_{\infty }+\frac{\varepsilon_{s}-\varepsilon_{\infty }}{1+{\left(j\omega \tau \right)}^{1-\alpha }},$$

where the purities and dielectric permittivity values of methanol, ethanol (Stanlab Sp. J., Lublin, Poland), distilled water at 20 ± 1 °C and the value of the *α*-parameter are presented in Table 1.

### 2.2. Simulations and Measurement Method

**Figure 1:**
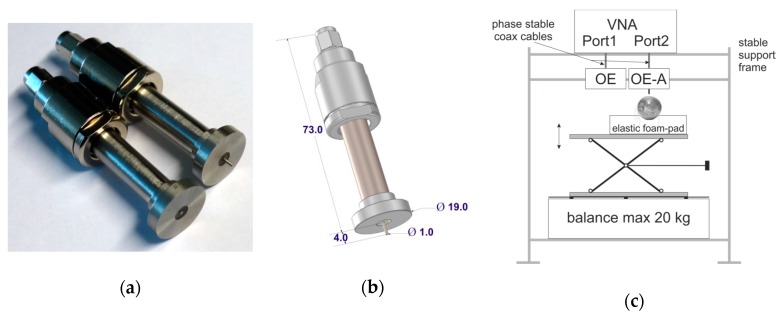
Tested coaxial probes and the elements of the measurement setup; (**a**) a coax OE probe (left) and a coax OE-A (right); (**b**) respective dimensions of the probes (in mm); and (**c**) experimental setup.

Two kinds of probes were used in this work: a coax OE probe and a coax OE-A (Figure 1a left and right, respectively). They were machined from the stainless steel. The dimensions (Figure 1b) were adjusted to have a 50 Ω impedance line between the SMA connector Pasternack PE4403 (Pasternack Enterprises, Inc., Irvine, CA, USA) and the ending fixed in a bead made of epoxy-resin. The dielectric constant of the epoxy resin was 3.8 ± 0.1 and was measured by the Agilent impedance analyzer E4980A with the 16451B dielectric test fixture.

The measurement setup consisted of a VNA (Anritsu VectorStar 10 MHz–20 GHz), a set of calibration and verification materials (calibration—air, distilled water and methanol, verification—ethanol) and a measurement fixture with a balance that assured 0.1 kg accuracy, which gives in conversion ±1 N force accuracy. The force put on the apple by the probe was equal to 10 ± 1 N. The whole setup was placed in a stable support frame as presented in Figure 1c.

**Figure 2:**
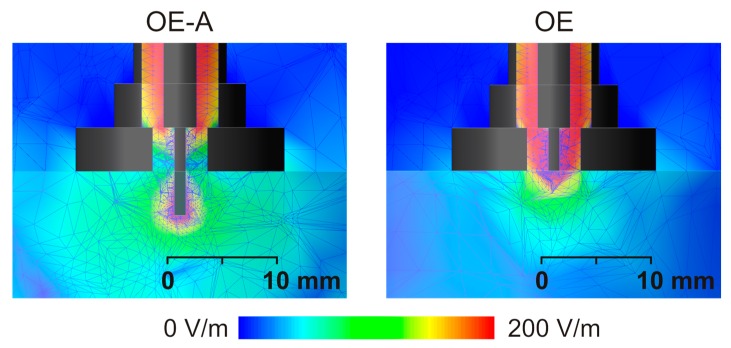
EMPro FEM simulations of electrical field penetration depth in distilled water for the frequency of 1.5 GHz.

Before measurements, numerical simulations for distilled water were performed using EMPro digital simulation software pack from Keysight (formely Agilent) (Figure 2). The mesh is made automatically by EMPro which chooses the most optimal mesh density, therefore, the Nyquist criterion was fulfilled. Absorbing boundary conditions were applied in simulations. Simulation results proved that the electrical field penetration depth generated by the applied OE-A probe is bigger than for OE probe (Figure 2). Imaginary part of distilled water electrical permittivity and electrical conductivity are smaller as compared to apples. Therefore, similar simulations were also performed for materials with higher conductivity, up to 0.8 S/m. Obtained results showed that electric field penetration depth was practically the same as presented in Figure 2 for distilled water.

During the measurements the probes were connected to the VNA through phase-stable cables (Anritsu 3671KFKF50-60). Each apple was measured six times with each probe, paying special attention to have no air gap between the probe flat ending and the apple. This was sometimes not possible because of the apple shape. Apple is a solid material with a curved surface and that prevents a good contact of the OE probe [20]. Therefore, two from six measurements were eliminated automatically as outliners by a dedicated MATLAB procedure. The outliner elimination procedure tested only the real part of the dielectric permittivity at the arbitrary chosen frequency of 100 MHz, where a flat shape of the spectrum begins. Having *n* curves, the outliner *i* curve was selected by calculating *n* standard deviations for the combination of *n* sets, each without one elementary curve. The minimal standard deviation from the calculated *n* values, indicated the curve that should be eliminated, leaving *n –* 1 curves. The procedure was applied again to eliminate another outliner curve. The same procedure of outliers elimination was repeated to reduce the number of apples from 10 to 7 in each series to make the measurement series representative. Each frequency scan included 1998 points in a logarithmic scale from 10 MHz–20 GHz, 600 points per decade. If the frequency was 300 Hz, a single measurement was performed.

**Figure 3:**
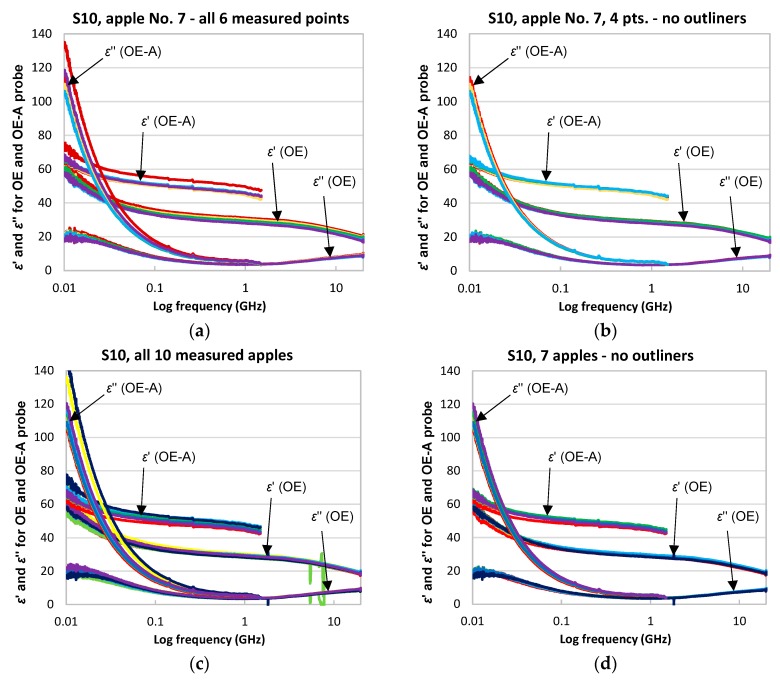
The scatter of data of recorded $\varepsilon_{r}^{*}$ spectra for the measurement series S10 collected with the OE and the OE-A probes, and the results of the applied elimination of outliers: (**a**,**b**) for the apple 7 in series S10; (**c**,**d**) for all apples in series S10.

**Figure 4:**
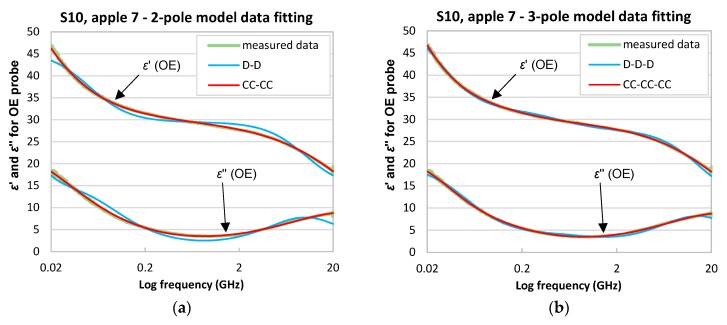
Results of the best and the worst data fitting of dielectric spectra of $\varepsilon_{r}^{*}$ collected with the OE probe for series S10, apple No. 7 for; (**a**) two-pole and (**b**) three-pole models.

**Figure 5:**
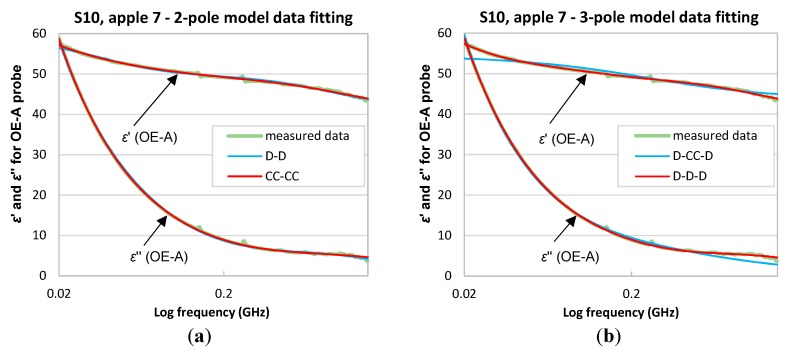
Results of the best and the worst data fitting of dielectric spectra of $\varepsilon_{r}^{*}$ collected with the OE-A probe for series S10, apple No. 7 for; (**a**) two-pole and (**b**) three-pole models.

Totally there were 11 measurement series S00–S10, each with 10 apples. After the series S00–S04 performed on five successive days, the time distance between measurements was increased because no significant differences in dielectric spectra were found (see Figure 3, Figure 4 and Figure 5).

The apples were measured with skin not removed so as to assure nondestructive measurements. This condition was not met in the case of the OE-A probe. The use of an OE-A probe involves the damage of the tested apples, but these tests give a good indication of the general quality of all the other apples. The measurement points were chosen in the middle height of an apple, between the stem and the blossom end.

### 2.3. Calibration of the Dielectric Probes

Before starting each measurement series, the coaxial probes were calibrated on air, water, and methanol according to the procedure described by Bao et al. [21]. This reference shows the proof that the S11 element of the scattering matrix measured at the input of VNA is related to the dielectric permittivity of the sample material at the tip of the OE probe in the form of the following bilinear equation:

(2) $$S_{11}=\frac{c_{2}+c_{3}\varepsilon_{r}^{*}}{c_{1}+\varepsilon_{r}^{*}}$$

where the calibration constants $c_{1},c_{2},\;\mathrm{and}\;c_{3}$ are complex numbers determined in the open-water-liquid (OWL) calibration [19] using air, distilled water, and methanol as the calibration media. The OWL calibration was chosen because reliable shorting the OE-A probe is rather difficult. Typical calibration of OE probe includes open-water-short (OWS) procedure, where shorting is done with the help of an indium foil [22].

Rewriting the Equation (2) for air, water and methanol gives:

(3) $$\left[\begin{array}{ccc} S_{11}^{O} & -1 & -1\\ S_{11}^{W} & -1 & -\varepsilon_{r,W}^{*}\\ S_{11}^{M} & -1 & -\varepsilon_{r,M}^{*} \end{array}\right]\left[\begin{array}{c} c_{1}\\ c_{2}\\ c_{2} \end{array}\right]=\left[\begin{array}{c} -S_{11}^{O}\\ -\varepsilon_{r,W}^{*}S_{11}^{W}\\ -\varepsilon_{r,M}^{*}S_{11}^{M} \end{array}\right]$$

where the respective superscripts *O*, *W*, *M* stand for open (the measured material is air), distilled water, and methanol, the scattering parameters $S_{11}^{O}$, $S_{11}^{W}$, $S_{11}^{M}$ are measured by VNA when the OE or OE-A probe measures air, distilled water, or methanol, $\varepsilon_{r,W}^{*}$ and $\varepsilon_{r,M}^{*}$ represent complex dielectric permittivity of distilled water and methanol. The value of dielectric permittivity of air $\varepsilon_{r,O}^{*}=1$ and therefore the element in the first row of the rightmost vector of the Equation (3) is $-S_{11}^{O}$. The Equation (3) must be solved for each frequency in the scanned range 10 MHz–20 GHz. The validation of the performed calibration was done with the dielectric measurements of ethanol. The calculated spectra of real and imaginary parts of ethanol dielectric permittivity were almost the same for the OE and OE-A probes in the frequency range 10 MHz–1.5 GHz. The relative difference for the Debye parameters were smaller than 2%.

Having determined the parameters $c_{1},c_{2},\;\mathrm{and}\;c_{3}$ from OWL calibration, the dielectric permittivity of the measured material $\varepsilon_{r}^{*}$ was calculated from the apple measured scattering parameter $S_{11}^{apple}$ using the rearranged form of the Equation (2):

(4) $$\varepsilon_{r}^{*}=\frac{c_{1}S_{11}^{apple}-c_{2}}{c_{3}-S_{11}^{apple}}$$

### 2.4. Fitting Data to Multi-Pole Relaxation Model

The spectrum of $\varepsilon_{r}^{*}$ was determined by fitting real and imaginary data calculated from Equation (4) to the three-pole relaxation model that includes the material conductivity (σ) element:

(5) $$\varepsilon_{r}^{*}=\varepsilon_{\infty }+\overset{n}{\underset{i=1}{\sum }}\varepsilon_{i}+\frac{\sigma }{j2\pi f\varepsilon_{0}}\;\varepsilon_{i}=\frac{\Delta \varepsilon_{fi}}{1+\frac{jf}{f_{fi}}}\;\mathrm{Debye}\;\mathrm{type}\;\mathrm{or}\;\varepsilon_{i}=\frac{\Delta \varepsilon_{fi}}{1+{\left(\frac{jf}{f_{fi}}\right)}^{\alpha }}\;\mathrm{Cole}-\mathrm{Cole}\;\mathrm{type}$$

where $\varepsilon_{\infty }$ is infinite relative permittivity; $\varepsilon_{0}$ ($8.85\times 10^{-12}$ F/m), σ(S) stand for air absolute permittivity and low frequency electrical conductivity; $\Delta \varepsilon_{fi}$ and $f_{fi}$ are at each next pole $\Delta \varepsilon_{fw}$, $\Delta \varepsilon_{bw}$ and $\Delta \varepsilon_{MW}$ stand for the real part dielectric permittivity decrease resulting from the associated relaxation process at $f_{fw}$ (for free water), $f_{bw}$ (for bound water), or $f_{MW}$ (for Maxwell–Wagner interphase effects) frequency (Hz); f(Hz) is the frequency of the applied electrical field. These values were determined by a nonlinear fitting procedure developed in MATLAB, firstly using the vector fitting approach [23] to determine the preliminary values of the Debye model parameters. Then, the preliminary values are refined using a full nonlinear least-squares fitting implemented with the MATLAB function lsqnonlin. The part: $\overset{n}{\underset{i=1}{\sum }}\varepsilon_{i}$ of Equation (5) consists of two or three poles. Each of them can be expressed by Debye (D) or Cole-Cole (CC) model, so there are eight different combinations for three-pole and 4 combinations for two-pole model. Therefore, $\varepsilon_{r}^{*}$ can be fitted by 12 different models.

The measurements were performed for the OE and OE-A probes in the range of 10 MHz–20 GHz and 10 MHz–1.5 GHz, respectively. Two- and three-pole models were fitted to the measurements data obtained for both, OE and OE-A probes (Table 2).

Apple tissue is a heterogeneous material containing water, dissolved organic molecules, macro- molecules, ions, and insoluble matter. The constituents are highly organized in cellular and subcellular structures forming macroscopic elements and soft and hard tissues. Their dielectric properties will, thus, reflect contributions to the polarization from both structure and composition [24].

## 3. Results and Discussion

### 3.1. Dielectric Permittivity Spectra of Apples

Figure 3a,b presents example frequency spectra of $\varepsilon_{r}^{*}$ calculated from the Equation (4), for the OE and OE-A probes in the range of 10 MHz–20 GHz and 10 MHz–1.5 GHz, respectively, each for six measurement points in the apple No. 7, series 10, of the real (upper part) and imaginary (lower part) of $\varepsilon_{r}^{*}$, for the OE and OE-A probes, respectively. Above 1.5 GHz the OE-A probe generates higher propagation modes and the applied calibrations and calculations presented by the Equations (3) and (4) are not sufficient.

Generally, it was found that, for each measurement point of an individual apple, the spectra of the dielectric permittivity differed, although the OE and OE-A probes were pressed against the apple with the same force and great care was taken to keep the flat ending of the OE and OE-A probes in parallel to the apple surface, which was not flat. The discrepancies were probably due to individual differences between apples, and an imperfect flat interface between the flat sensor and the spherical shape the apple. Therefore, two of six measurement points at each measured apple were eliminated as outlier points to minimize the measurement error.

Having four selected frequency spectra of $\varepsilon_{r}^{*}$ for each apple, the mean value of them was calculated taking ten mean frequency spectra for each measurement series S00–S10, each one for an individual apple in a series. A similar outlier elimination procedure was applied for ten apples in each series so as to finally have seven apples. The outlier elimination procedure decreased the standard error of measurement at least by a factor of 2.

The differences between real (and imaginary) parts of the spectra measured with the OE and OE-A probes, presented in Figure 3, are caused by an apples’ structure. The skin and flesh of apples have different electric parameters and the apple is not a homogenous material (in contrast to, e.g., ethanol). The apples were measured with skin not removed. The OE-A probe has a larger measurement volume than the OE probe. Therefore, the electric field in the case of both probes penetrate the outer layer of an apple (the skin) and flesh of the apple, but the OE-A probe has an antenna which is inserted into the flesh and the volume penetration ratio flesh/skin is larger for the OE-A probe than the OE probe. This explains such differences in the measured permittivity spectra. However, the OE-A probe had the upper frequency limit of 1.5 GHz as compared to 20 GHz for the OE probe.

### 3.2. Multipole Model Fitting Comparison

The multipole model fitting was performed for apples in series S00–S10. Data collected using the OE and OE-A probes were fitted by two- and three-pole models. Table 2 presents the root-mean-square-errors (RMSE) of multipole data fitting procedure for S10 apple No. 7.

In any case the best fit was obtained for models with Cole-Cole poles and the worst with Debye poles (Table 2). Only for OE-A data fitted by the three-pole model the worst fit was obtained using D-CC-D and the best for D-D-D model, but CC-CC-CC also gives very good results. The sequence of poles showed in Table 2 corresponds to $f_{fw}$, $f_{bw}$, $f_{MW}$, from left to right, for the three-pole model and $f_{bw}$, $f_{MW}$ for the two-pole model.

It occurred that the three-pole models gave better fit for OE probe that the two-pole models, while, for the OE-A probe, the two-pole models were generally better. This corresponds to the fact that, in the case of the OE-A probe, the limited frequency range did not enable an accurate fitting of the high-frequency free-water relaxation pole.

Figure 4a,b and Figure 5a,b present the best and the worst two- and three-pole model data fitting for data collected using the OE and OE-A probes, respectively. 

### 3.3. Relations between Quality and Multipole Relaxation Model Fitting Parameters

**Figure 6:**
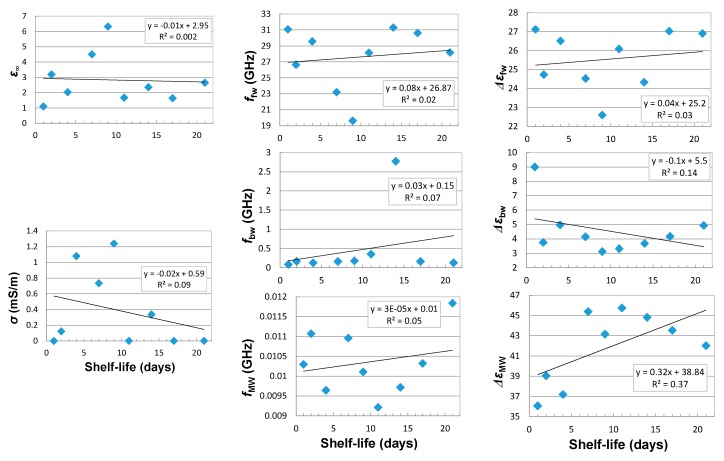
Correlation between the parameters of CC-CC-CC multipole relaxation model and the shelf-life of the tested apples determined by the OE probe (assuming the influence of electrical conductivity *σ* (mS/m) and three dielectric dispersion effects from Maxwel-Wagner—*MW*, bound water—*bw* and free water—*fw*).

**Figure 7:**
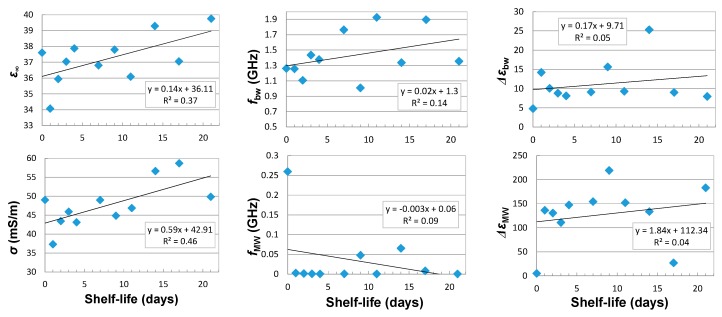
Correlation between the parameters of the multipole model and the shelf-life of the tested apples determined by the OE-A probe (assuming the influence of electrical conductivity *σ* (mS/m) and two dielectric dispersion effects from Maxwel-Wagner—*MW* and bound water—*bw*).

There was no correlation found for the OE probe between the apple shelf-life and the parameters of the multipole model described by Equation (5) with three dispersion effects described by the Cole-Cole model (Figure 6). This is probably due to the presence of apple skin, which masks the apple-flesh properties that are most important for the shelf-life period. This is confirmed by slightly better correlations received for the OE-A probe, in the case of electrical conductivity σ, bound water relaxation frequency $f_{bw}$, and high-frequency dielectric parameter $\varepsilon_{\infty }$ (Figure 7). The respective multipole relaxation model included only two dispersion effects (as the result of 1.5 GHz frequency limit for the OE-A probe) caused by the bound water and the interphase polarization (Maxwell-Wagner effects).

**Figure 8:**
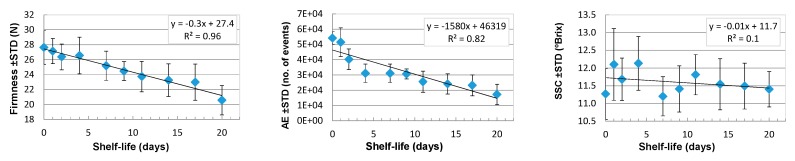
Mean values of firmness - left, acoustic events (AE)—middle, and soluble solids content (SSC)—right variability in apples in each test during the 21-day long shelf-life.

The temporal variability of apple physiochemical parameters in each series is presented in Figure 8. No correlation was found for soluble solids content and the shelf-life, but the apple firmness measured by the puncture and acoustic emission methods showed good correlations ($R^{2}>0.8$).

**Figure 9:**
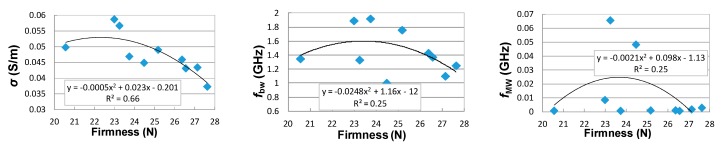
Relation between firmness of the tested apples and selected multipole model parameters (low frequency electrical conductivity *σ* (mS/m)—left, relaxation frequency of bound water effect *bw* —middle and relaxation frequency of Maxwell-Wagner effect *MW*—right) for $\varepsilon_{r}^{*}$ measured with the OE-A dielectric probe.

Correlations between dielectric spectrum parameters measured with OE-A probe and shelf-life is better than for the OE probe (Figure 6 and Figure 7). The exceptions are the Δ*ε*_bw_ and Δ*ε*_MW_ parameters, which show better correlations for measurements performed using the OE probe. Figure 8 presents quite good correlations of firmness and acoustic events with shelf-life, with $R^{2}=0.96$ and $R^{2}=0.8$, respectively. Correlation between soluble solid content and shelf-life is much worse (*R*^2^ < 0.1). Taking into account poor correlations between parameters of the multipole dielectric dispersion model, apple quality parameters, and shelf-life (small *R*^2^), finding a good correlation between dielectric properties and quality parameters is difficult. Figure 9 presents correlations of σ, $f_{fw}$, and $f_{MW}$ with firmness. As can be seen, the most reliable correlation can be found for low-frequency electrical conductivity σ and firmness, where the second-degree polynomial is fitted with $R^{2}≅0.7$.

## 4. Conclusions

Due to the diversity of apples in a harvested bunch it was necessary to eliminate outliers, i.e., eliminate measurement points in individual apples with outlying results and also eliminate apples with outlying mean-measurements in each measurements series. Outlier elimination decreased the standard deviation of measurement by about two times in each case.

Two- and three-pole data fitting models with different combinations of poles were tested. The best fitting results were obtained using two- and three-pole Cole-Cole models for OE-A and OE probes, respectively.

The dielectric spectrum parameters measured with the probe with an antenna showed quite good correlation of the multipole model parameters with the apples’ shelf-life quality parameters, for example, firmness, unlike the spectrum parameters measured with the OE probe. This may have been caused by the greater penetration depth of the electric field in the apples. Especially, the low-frequency conductivity was strongly negatively correlated with the firmness of the apples. However, the OE probe measures apples with the skin undisturbed and the lack of clear correlations with shelf-life parameters proves that the skin has the greatest influence on the apple dielectric response and has practically the same dielectric properties during apples’ shelf-life.

The performed dielectric measurements of the apples confirmed earlier reports that the tests should be conducted on fruits without skin. It seemed that the temporal variability of 21 days was too short to report distinct variability of apples’ dielectric permittivity parameters, using nondestructive methods, during the process of their maturity.

## Figures and Tables

**Table 1:** Purities and dielectric permittivity values of methanol, ethanol (Stanlab Sp. J., Poland) and distilled water at 20 ± 1 °C used in calibration of the OE and OE-A probes [19].

	Purity	*ε_s_* (-)	*ε_∞_* (-)	*τ* (ps)	*α* (-)
Water	Electrical conductivity <2 μS/cm	80.18	5.54	9.57	0.00
Ethanol	Min. 99.7%	25.16	4.54	193.98	0.14
Methanol	Min 99.8%	33.65	5.65	56.36	0.00

**Table 2:** RMSE for two- and three-pole fit procedure for data collected using the OE and OE-A probes.

3-Pole Model	RMSE		2-Pole Model	RMSE	
	OE	OE-A		OE	OE-A
D-D-D	0.3489	0.1654	D-D	0.8876	0.2984
CC-D-D	0.1143	0.3241	CC-D	0.4047	0.2071
D-CC-D	0.1810	0.8363	D-CC	0.3733	0.1808
D-D-CC	0.1835	0.6360	CC-CC	0.1281	0.1796
CC-CC-D	0.0967	0.1942			
CC-D-CC	0.0955	0.2271			
D-CC-CC	0.1295	0.2642			
CC-CC-CC	0.0951	0.1894			

## Abbreviations

OE	Open-ended
OE-A	Open-ended with antenna
D	Debye
CC	Cole-Cole
SSC	Soluble solid content
AE	Acoustic events
STD	Standard deviation
FEM	Finite Element Method
RMSE	Root-Mean-Square Error
VNA	Vector Network Analyzer
OWL	Open-Water-Liquid calibration
OWS	Open-Water-Short calibration
fw	Free water
bw	Bound water
MW	Maxwell-Wagner
